# Using Localization Microscopy to Quantify Calcium Channels at Presynaptic Boutons

**DOI:** 10.21769/BioProtoc.5049

**Published:** 2024-08-20

**Authors:** Brian D. Mueller, Sean A. Merrill, Lexy Von Diezmann, Erik M. Jorgensen

**Affiliations:** 1 School of Biological Sciences, University of Utah, Salt Lake City, UT, USA; 2 Howard Hughes Medical Institute, Salt Lake City, UT, USA; 3 Dept. of Genetics, Cell Biology, and Development, University of Minnesota, Minneapolis, MN, USA

**Keywords:** *C. elegans*, Super-resolution microscopy, Quantitative localization microscopy, Calcium channels, Pre-synapse, Neuroscience

## Abstract

Calcium channels at synaptic boutons are critical for synaptic function, but their number and distribution are poorly understood. This gap in knowledge is primarily due to the resolution limits of fluorescence microscopy. In the last decade, the diffraction limit of light was surpassed, and fluorescent molecules can now be localized with nanometer precision. Concurrently, new gene editing strategies allowed direct tagging of the endogenous calcium channel genes—expressed in the correct cells and at physiological levels. Further, the repurposing of self-labeling enzymes to attach fluorescent dyes to proteins improved photon yields enabling efficient localization of single molecules. Here, we describe tagging strategies, localization microscopy, and data analysis for calcium channel localization. In this case, we are imaging calcium channels fused with SNAP or HALO tags in live anesthetized *C. elegans* nematodes, but the analysis is relevant for any super-resolution preparations. We describe how to process images into localizations and protein clusters into confined nanodomains. Finally, we discuss strategies for estimating the number of calcium channels present at synaptic boutons.

Key features

• Super-resolution imaging of live anesthetized *C. elegans.*

• Three-color super-resolution reconstruction of synapses.

• Nanodomains and the distribution of proteins.

• Quantification of the number of proteins at synapses from single-molecule localization data.

## Graphical overview


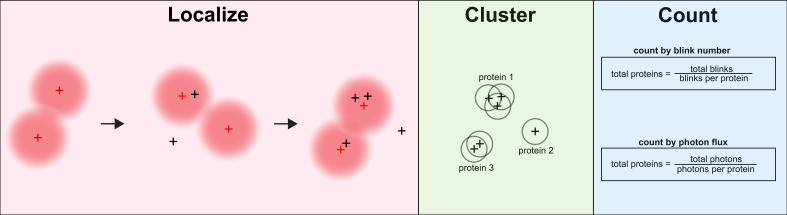


**Emissions from blinking fluorophores are collected, and 3D point spread functions are fit to calculate emitter positions.** Emitters are clustered by position and resolution. These clusters represent individual proteins or clumps of proteins. Based on single protein statistics, the number of proteins at any position is approximated by the number of blinks at a site or the total photon flux per site.

## Background

Proteins’ scale is in the order of nanometers; for example, GFP is approximately 4 nm across. However, due to the diffraction limit of light, collecting and focusing the light from GFP onto a camera sensor will, at best, produce an Airy disk with a diameter of 440 nm. To understand the distribution of proteins in cells, microscopy methods that can bypass the diffraction limit of light are required. Resolution is the spatial limit at which the certainty of resolving two nearby proteins fails; thus, high spatial resolution becomes particularly important for small subcellular structures like synapses [1]. Synapses contain highly specialized domains, such as the active zone, but are just a few hundred nanometers in diameter. These highly organized domains allow rapid and reliable neurotransmission [2–4]. To understand how these domains work, the underlying proteins and their nanoscale organization relative to each other must be determined.

Super-resolution methods that achieve resolutions below 100 nm can be divided into stimulated emission microscopy (for example, STED) or localization microscopy (for example, PALM and STORM). Stimulated emission is a scanning method that, in essence, reduces the diameter of the laser beam. Localization microscopy illuminates the whole sample and collects a 2D image of fluorescence on a camera face but limits the number of molecules that contribute to any single frame of the movie so that they appear as single blinks of light. Next, a modeled 3D-point spread function is fit to the blinks of light to reconstruct an image in x, y, and z [5–8].

The coordinate data produced from fitting a 3D-point spread function to the signal from each emitter has a precision determined by the Cramér-Rao lower bound (reviewed in von Diezmann et al. [9]). In contrast, the entire set of coordinate data achieves a resolution that can be determined by several methods. Fourier ring analysis is one common method to determine the resolution that reflects both localization precision and how well a set of localizations samples the underlying structure [10]. By this metric, we typically obtain datasets with 40 nm resolution from live worms. Given that ion channel complexes are ~10 nm in diameter, these data fall short of resolving single channels.

Beyond single proteins, we aim to determine the organization of two different proteins relative to one another. The spatial distributions of distinct proteins can be related in four ways: the proteins can be organized into a single coincident domain, overlapping domains, or adjacent domains, or be completely disjointed. Critically, the *borders* of protein domains, like the borders of a country, describe the essential information. While more difficult to analyze than an image, coordinate data can more completely describe these spatial relationships.

Here, we demonstrate multicolor super-resolution imaging in live anesthetized *C. elegans* of directly tagged and labeled endogenous proteins. Further, we discuss quantifying proteins and analyzing images from 3D single-molecule localization microscopy data. This protocol describes how to collect and analyze data from individual synapses, to count the number of ion channels, and determine the spatial relationships between nanodomains of different proteins.

**Figure 1.**
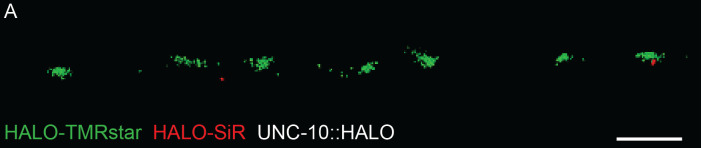
HALO-TMRstar saturates UNC-10::HALO after 60 min. L4 animals were incubated with 5 μM HALO-TMRstar (Green) for 60 min. After initial staining, animals were allowed to recover on a nematode growth media (NGM) plate with OP50 bacteria for 1 h. The animals were stained again in 5 μM HALO-SIR (Red) for 60 min and then allowed to recover for 4 h. Synaptic regions near the pharynx were imaged by localization microscopy. Scale bar = 1 μm.

For counting single proteins, the use of endogenous labels vs. antibodies has two advantages. The first is saturation. The TMR dye saturates endogenous proteins tagged with HALOtag in one round of staining (Figure 1). Therefore, the number of proteins can be quantified without caveats from epitope availability or binding affinities of antibodies [11]. The second is precision. Antibodies introduce an additional ~20 nm of uncertainty into localization due to their size. By contrast, the size of self-labeling enzyme tags is approximately 4 nm. One drawback of live cell imaging vs. antibodies on fixed samples is that oxygen-scavenging buffers cannot be used, leading to increased photobleaching (and thus lower photon counts or fewer localizations). Given these considerations, our protocol produces a reconstruction with sufficient localizations and resolution considering the typical size and separation of neuronal protein microdomains [12–14].

To determine if two protein microdomains are associated, Pearson’s or Mandel’s correlation coefficients are commonly used to determine colocalization in fluorescence microscopy [15]. These tests rely on diffraction-limited signals to create overlapping signals. However, for super-resolution methods, as spatial resolution approaches the size of a protein, the correlation coefficients in these tests will approach zero. For these reasons, it is preferable to analyze the data using nearest neighbor values. Importantly, nearest neighbor analysis distinguishes domains that are coincident, domains that do not overlap but are adjacent, domains at fixed distances, and domains with no relationship. In contrast to nearest neighbor measurements, the spatial relationship of localizations to a center axis or biological landmark has been successful at describing the underlying biology [1,16]. In the following protocol, we describe how to count the number of proteins in a nanodomain, how to measure the size of the domain, and how to determine relationships between protein domains based on localization coordinates.

For this study, we used a Bruker Vutara SRX352. In brief, this is a super-resolution microscope that uses wide-field illumination of the sample. Wide-field illumination activates fluorophores and collects light from an entire vertical column; thus, thin samples or samples positioned with regions of interest near the objective are crucial for reducing background emissions. The microscope uses a proprietary optical biplane that splits the focal plane by ~700 nm in Z-space onto the camera face. Biplane imaging permits improved axial resolution during reconstructive microscopy and provides 1.2 μm of optical thickness at a single z-position. Further, this microscope is capable of 3-color super-resolution imaging with 488, 561, and 641 nm excitation lines. These laser lines are paired with emissions filters for green (BP497-538), orange (BP570-629), and red (BP647-739) fluorophore imaging. Although we describe the specific routine for a Vutara SRX352, in principle the steps listed here are applicable to any super-resolution microscope.

Here, Sections 1–4 deal with the details for mounting and imaging *C. elegans* nematodes, and Sections 4–8 concern imaging and analysis of localizations.

## Materials and reagents

**Biological materials**

1. *C. elegans* strain EG9617 (Jorgensen Lab, *elks-1::Skylan-S, egl-19::SNAP IV; unc-2::HALO X*)
2. *C. elegans* strain EG9667 (Jorgensen Lab, *elks-1::Skylan-S, egl-19::SNAP IV; unc-68::HALO V*)
3. Nematode growth media (NGM) plates seeded with OP50 (see Wormbook.org)

**Reagents**

1. DMSO (Millipore Sigma, catalog number: D2650-5X5ML)
2. JF549cp-SNAPtag Ligand (Janelia Labs), store freeze-dried at -80 °C. Available by request from: https://janeliamaterials.azurewebsites.net/
3. JF646-HALOtag Ligand (Janelia Labs), store freeze-dried at -80 °C. Available by request from: https://janeliamaterials.azurewebsites.net/
4. Sodium azide (NaN_3_) (Millipore Sigma, catalog number: S2002)
5. Agarose (Gold Biotechnology, catalog number: A-201-1000)
6. NaCl (Millipore Sigma, catalog number: S5886-5KG)
7. K_2_HPO_4 _(Millipore Sigma, catalog number: P8281-500G)
8. Na_2_HPO_4 _(Fisher Scientific, catalog number: S373-3)
9. MgSO_4 _(Millipore Sigma, catalog number: M2643-500G)

**Solutions**

1. M9 buffer (see Recipes)
2. 25 mM sodium azide in M9 (see Recipes)
3. 4% agarose in M9 (see Recipes)

**Recipes**

1. **M9 buffer**
  **Table:** 

Reagent	Final concentration	Amount
K_2_HPO_4_	22 mM	3 g
Na_2_HPO_4 _	34 mM	6 g
NaCl	86 mM	5 g
MgSO_4 _(1 M)	1 mM	1 mL
H_2_O	n/a	1,000 mL
Total	n/a	1,000 mL
  M9 has a shelf-life of one year. Dispose if fungal or bacterial contamination occurs.
2. **25 mM sodium azide in M9**
  **Table:** 

Reagent	Final concentration	Amount
Sodium azide (powder)	25 mM	16.26 mg
M9 buffer	n/a	10 mL
Total	n/a	10 mL
  Store at -20 °C in single-use aliquots to avoid repeated freeze-thaw cycles. The paralytic properties of sodium azide solution begin to degrade after one year in storage.
3. **4% agarose in M9**
  **Table:** 

Reagent	Final concentration	Amount
Agarose	4%	2 g
M9 buffer	n/a	50 mL
Total	n/a	50 mL
  Heat in the microwave until dissolved. Store at 4 °C in aliquots to avoid evaporation from heating cycles. Agarose aliquots have a shelf-life of one year. Dispose if evaporation or fungal or bacterial contamination occurs.

**Laboratory supplies**

1. 1.5 mL microcentrifuge tubes (Life Science Products, model: Omniseal, catalog number: M-1700C-1M)
2. Pasteur pipettes (Fisher Scientific, catalog number: 13-678-20B)
3. Microscope slides (Fisher Scientific, model: Gold Seal, catalog number: 3048)
4. Zeiss 1.5 high-performance coverslips (Zeiss, catalog number: 474030-9000-000)
5. Worm pick (see Wormbook.org)

## Equipment

1. Vutara SRX 352 (Bruker)
2. Benchtop centrifuge (Thermo Fisher, model: mySPIN6, catalog number: 75004061)
3. Heat block set to 95 °C (e.g., Eppendorf, model: ThermoMixer F1.5, catalog number: 5384000020)

## Software and datasets

1. Vutara SRX (7.00.00rc39, 2020)
2. MATLAB (R2022a)
3. Microsoft Excel

## Procedure

- **Synchronize worms by staging embryos**
  1. Day 1
    - At 9:00 am, use a worm pick to move 20 young adult animals onto a new NGM plate seeded with OP50; do this three times to create three plates total. This procedure takes approximately 10 min.
    - At 5:00 pm, move the 20 adult animals from each plate onto new NGM plates seeded with OP50 to create three additional plates.
  2. Days 2–3
    - At 9:00 am, move the 20 adult animals from each plate onto new NGM plates seeded with OP50 to create three additional plates.
    - At 5:00 pm, move the 20 adult animals from each plate onto new NGM plates seeded with OP50 to create three new plates.
    - Move the original adult animals to fresh plates in the morning and evening for one more day.
- **Label SNAP and HALO**
  1. Day 4, evening
    - Inspect plates to select for broods with an abundance of L4 hermaphrodite animals. This is a synchronized plate.
    - Wash animals off the three synchronized plates using ~1 mL of M9 buffer and a glass Pasteur pipette. Place animals into a microcentrifuge tube. Worms will stick to the plastic of a micropipette, so use glass to move animals.
    - On a benchtop centrifuge, gently spin (10 s each) to remove bacteria, removing the supernatant and washing with 1 mL of M9 buffer.
    - After three washes, remove the supernatant and resuspend the animals in approximately 195 μL of M9 buffer.
    - Prepare JF dyes by resuspending at 1 mM in DMSO (e.g., 5 nmol of dye in 5 μL of DMSO). Move dye + DMSO suspension into the next dried dye aliquot to create a combination of dyes in suspension. This will create 5 μL of 1 mM JF646-HALOtag Ligand + JF549cp-SNAPtag Ligand dye.
    - Add dye + DMSO solution to worms in M9 buffer to create a 25 μM final concentration of dye. Mix gently by vortexing.
*Note: DMSO above 5% is toxic to worms.*
    - Put the tube with the lid in a freezer box on an orbital shaker and stain for 120 min at RT. Gently flick and spin down worms every 15 min to prevent worms from forming clumps on the bottom of the tube or becoming stuck on the sides of the tube. **It is essential that the dye and worms incubate in a dark place like a lidded freezer box.**
    - After 2 h, spin down the worm solution, aspirate off the supernatant, and wash four times with 1 mL of M9 buffer.
    - With a Pasteur pipette, transfer worms to at least three OP50-seeded NGM plates. Let the M9 droplet dry completely before inverting the plate. This should be performed in the dark (we use a drawer). After the droplet has dried, cap the plates and invert. Let the worms recover in a lidded freezer box for 10–12 h. It is important to not expose the stained worms to light. Ideally, the L4 to adult molt will occur during recovery to minimize background staining of the cuticle and gut.
*Note: L4 hermaphrodite animals can be identified by the absence of a vulva, which appears as a white crescent at the center of the ventral side of the animal under a typical dissection microscope. Starting with synchronized plates is perhaps the most crucial step of the protocol because the dye sticks to the cuticle of the animal. If adults are exposed to dye, they will have non-specifically labeled cuticles forever, which interferes with imaging of the nervous system. L4s are the final larval stage of the animal and must molt their cuticle before adulthood. Thus, if L4s are stained and then imaged as young adults, a molt must have occurred during recovery.*
- **Imaging preparation**
  1. Day 5, morning
    - Prepare a 4% agarose pad by heating an agarose aliquot in a 95 °C heat block and placing a small drop on a microscope slide. Then, flatten the pad with another microscope slide (Figure 2A).
    - Trim the agarose pad to about 10 mm × 5 mm using a microscope slide. The agarose pad should fit entirely under the coverslip (Figure 2B).
    - Add 2–4 μL of 25 mM NaN_3_ to the pad.
    - Use the worm pick to distribute the NaN_3_ drop evenly across the agarose pad. If NaN_3_ flows over the edges of the agarose pad, reduce the amount used.
    - Transfer 20 young adult worms to the agarose pad. Older animals may have been young adults during staining and will be highly fluorescent because they have not shed their cuticle.
    - Wait for animals to paralyze and straighten. This should take a few minutes. If the droplet of NaN_3_ is drying out, place the slide into a humidity chamber. Seal the pad with a coverslip. Then, seal the coverslip with nail polish and image (Figure 2B).
*Notes:*
*i. Do not image animals that have been in* NaN_3_ *for more than 60 min. The paralytic sodium azide causes neuron blebbing and disruption of cell morphology after approximately 1 h.*
*ii. Avoid air bubbles in the agarose pad or trim them.*
  **Figure 2.**
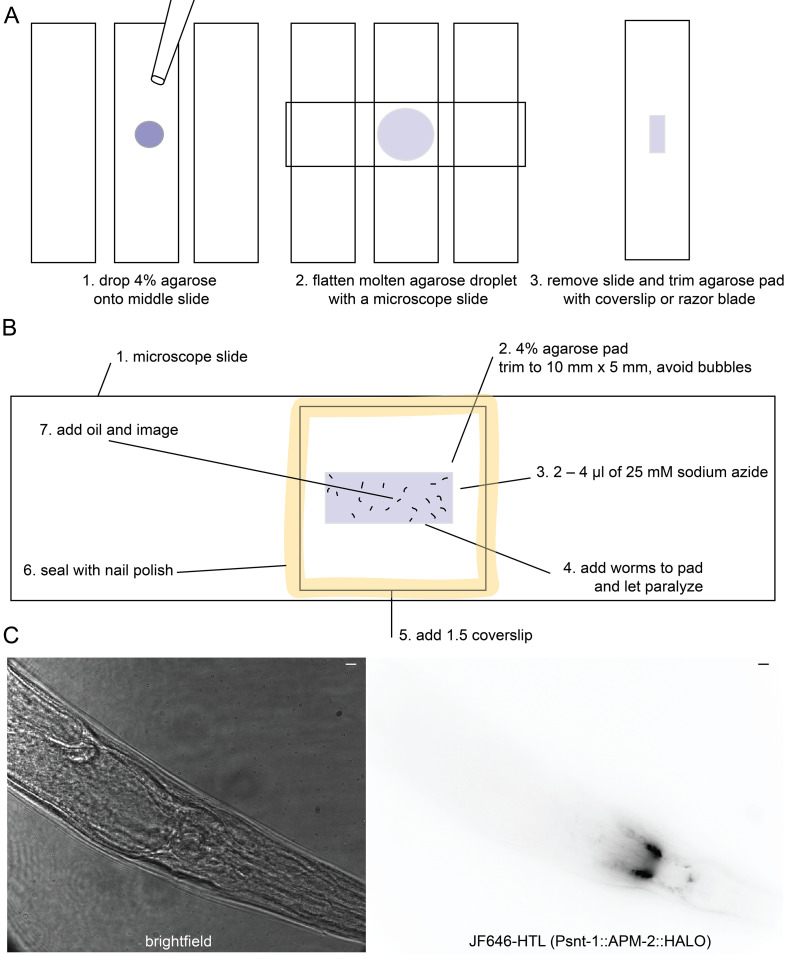
Creating a worm imaging pad and slide. (A) The agarose pad acts as a support for the worm sample so it is not crushed by the microscope slide, also immobilizing the animal. (B) Once sealed with nail polish, the worms should last for hours. However, the sodium azide paralytic causes cell blebbing and disruption of cell morphology after about an hour. (C) Left: Under the brightfield illumination of the Vutara SRX 352, the worms will appear as pictured. Right: The roll can be in part determined by the position of the ventral nerve cord exiting the neuropil if there is a fluorescent marker in the nervous system or position of the vulva. Scale bar = 5 μm.
- **Superresolution imaging of *C. elegans***
  1. Image worms by 3-color imaging
    - Organize laser lines in descending order of wavelength, e.g., 1) 646 nm, 2) 549 nm, 3) 488 nm. This reduces the photobleaching of the sample.
    - In widefield mode: Locate worms on the pad and coverslip.
    - In super-resolution mode:
      i. Use only extremely low laser powers (< 1%).
      ii. Set the exposure time to 300 ms.
      iii. Focus both imaging planes as much as possible on the dorsal nerve cord (Figure 2A).
      iv. **Turn lasers off as soon as possible. We try to have the laser on for under 10 s while focusing on the synapses we plan to image.**
    - After the nerve cord is in focus, reduce the exposure time to 30 ms and proceed to record. Tune laser powers for optimal blinking vs. the lifespan of the fluorophores. An example of a properly tuned laser power is shown in Figure 3B.
*Notes:*
*i. The best samples are worms that are oriented with their dorsal nerve cord tilted toward the objective.*
*ii. Be very cautious with the laser power while finding your sample. The worm is not in oxygen scavenging buffer; thus, fluorophores will bleach. To focus on the cord, use very low laser power and a 300 ms exposure time.*
*iii. Optimizing laser power while imaging: A laser power that is too low will not induce blinking of the fluorophores. A laser power that is too high will bleach the sample in a few frames. The user should find these limits and note them for their microscope. The localization counts over time displayed in Figure 3B is an example of an ideally tuned laser power.*
  2. Image worms—counting channels
    - Organize laser lines by starting with the protein you wish to quantify, followed by fiducial markers. Fiducial markers label a known structure, like the synaptic dense projection, and are used to spatially orient the experimental channel.
    - In widefield mode: Locate worms on the pad and coverslip.
    - In super-resolution mode:
      i. Use only extremely low laser powers (< 1%).
      ii. Set the exposure time to 300 ms.
      iii. Focus both imaging planes as much as possible on the dorsal nerve cord.
      iv. **Turn lasers off as soon as possible.**
    - After the cord is in focus, reduce the exposure time to 30 ms and proceed to record. Increase the laser powers for optimal blinking vs. lifespan of the fluorophores. This is typically between 4% and 12% with 200 mW lasers and the 40 μm biplane module. For counting emitters, the dyes should be completely bleached after imaging; this typically occurs within 4,000 frames.
*Notes:*
*i. The best samples are worms that are oriented with their dorsal nerve cord tilted toward the objective.*
*ii. Optimizing laser power while imaging: A power that is too low will not induce blinking of the fluorophores. A laser power that is too high will bleach the sample in a few frames. The user should find these limits and note them for their microscope. The localization counts over time displayed in Figure 3B is an example of an ideally tuned laser power.*
- **Localizing**
  1. Proceed to the *Localization* tab.
  2. Set the background threshold. This should be optimized to minimize background localizations (false positive) but not exclude real emitters (false negative). Adjust the background threshold to produce cutouts (colored boxes) around real signals but minimal cutouts in areas with no signal. An example of the effect of background thresholds is displayed in Figure 3C. For *C. elegans*, we find the ideal threshold to be between 3 and 5 and rely on denoising (detailed below) to remove false positives.
  3. Select images that demonstrate blinking by adjusting the region of interest frame (Probe x) slider. This is primarily to exclude frames where fluorophores are not blinking. Blinking can be determined by moving 10 frames forward and 10 backward (600 ms range). If emitters are turning on and off between those frames, then they are suitable to analyze.
*Notes:*
    - *At the beginning of imaging, all fluorophores will be active, and separate light flashes will not be visible. Blinking may not occur for a few hundred frames into the acquisition. Non-blinking frames should not be analyzed.*
    - *Background threshold should be optimized across the acquired frames.*
    - *Localization cutouts can be turned on in Expert Options under the* Localizations *tab.*
    **Figure 3.**
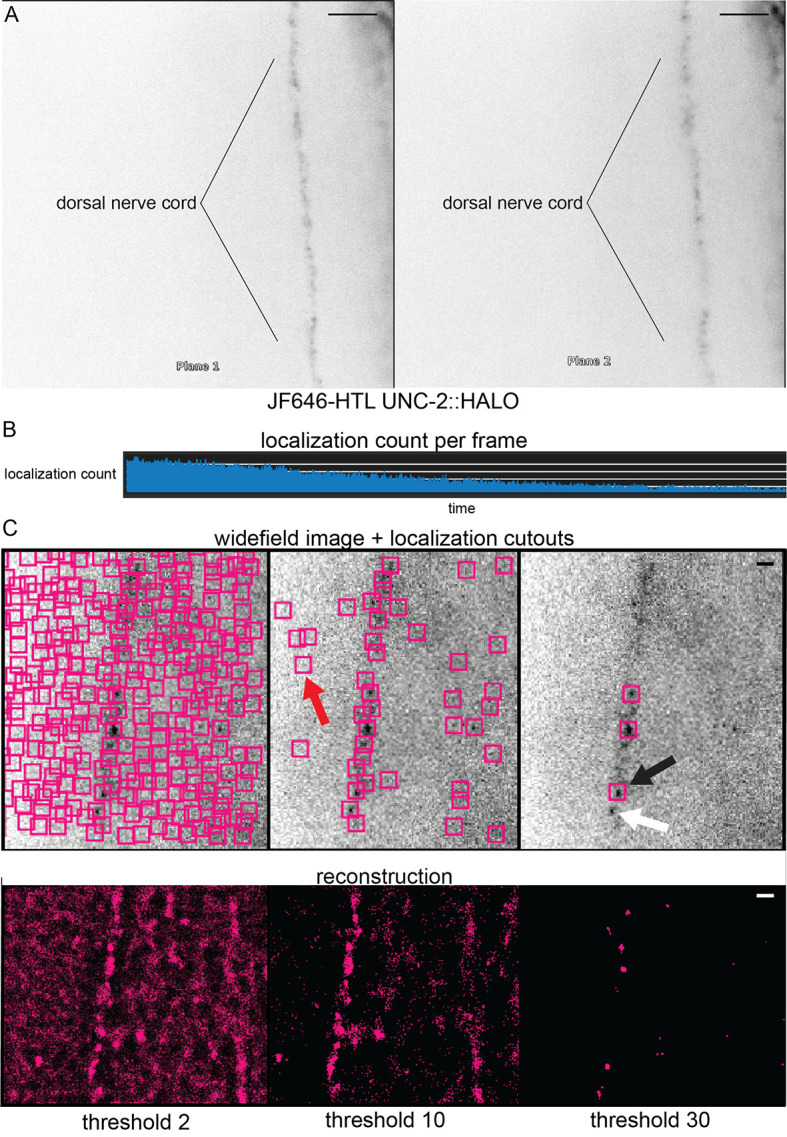
Biplane, blinking, and background thresholding. (A) The Vutara SRX simultaneously images in two focal planes (“biplane”), which splits the light path into two focal planes separated by about 700 nm. To focus on a structure like the dorsal nerve cord, it should be equally in focus on both planes—which ensures that the emitters are in between the two focal planes. Scale bar = 5 μm. (B) Graph of localization cutouts over time. At the start of imaging, there will be many fluorophores in an excitable state. However, as imaging progresses, fluorophores will begin to enter the dark state. This change can be confirmed by the drop in localization cutouts over time. Stochastic blinking is required for super-resolution and can be confirmed by the spikes and troughs in the number of cutouts over time. (C) Examples of background thresholding on image reconstruction of CaV2 in the dorsal nerve cord (background threshold = 2, 10, or 30). Upper: Magenta boxes represent successful PSF fitting. Red arrow shows an example of a false positive. Black arrow shows an example of a successful localization. White arrow shows an example of a false negative. Lower: Magenta points represent protein localizations. Scale bar = 1 μm.
- **Visualizing**
  Proceed to the *Visualization* tab.
  1. Discard localizations that are not accurate in the *Advanced Particle Filters* dialogue. For live worms, we set an arbitrary threshold of 50 nm radial precision and display localizations on the visualization plot as a 50 nm particle size (Figure 4A).
  2. Denoise the image using *Method: Mean Distance* and denoise by *Percent*. Lower the slider until most of the signal outside the cord is removed but the signal inside the cord appears unperturbed (Figure 4B).
  3. Save this image to the View Manager.
  4. For analysis of proteins at individual synaptic boutons, select a region of interest 700 nm radius from the center of an in-focus dense projection. Save this view in View Manager. Export the data and save it as a particle .csv file. Out-of-focus synapses and synapses with chromatic aberrations in the XY plane are discarded. Some chromatic aberrations will be present in the Z plane. Aim to analyze five synapses per animal from five different animals, with a total of 25 synapses analyzed. We recommend saving particle files ordered by “animal#_synapse#”, e.g., “3_1.csv.”
    **Figure 4.**
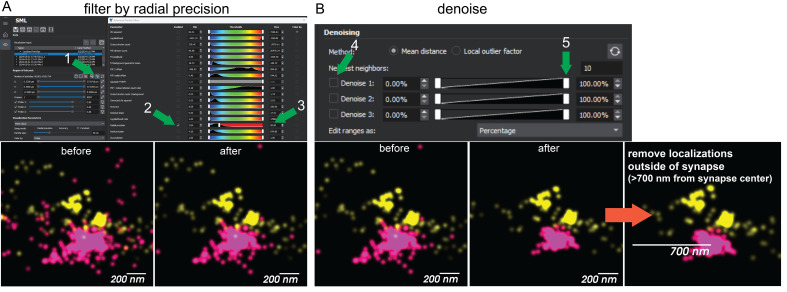
Data filtering. (A) First, reconstructions are filtered to exclude imprecise localizations. (B) Next, localizations are trimmed based on nearest neighbors using the denoising tool. Finally, a region of interest is drawn around a synapse to be analyzed to exclude localizations that cannot be within the synapse based on the size of the bouton. Numbering and green arrows show the click order for the user. Magenta = CaV2, Yellow = CaV1.
- **Cluster analysis**
  1. Click on *Advanced Statistics Dialogue* and go to the *Cluster Analysis* tab.
  2. Set the parameters for calling clusters based on the size of the protein and resolution of the image. See note.
  3. Click *Compute*.
  4. Export the resulting output as a .csv file.
*Note: We assume that multiple localizations within a close distance are the same channel blinking multiple times. That distance is defined by the size of the calcium channel and the resolution. Voltage-gated calcium channels are approximately 10 nm in size, and localizations worse than 50 nm in precision were discarded. Thus, any localizations within 60 nm could be from the same channel. Therefore, the* Maximum particle distance *is set to 60 nm. The Minimum particle count to form cluster is set to 2. An example of clustering is available in Figure 5A.*

## Data analysis

Mapping distributions of proteins from particle files (produced in section F):

1. Open MATLAB and load the following script into a command window.
https://github.com/bdmscience/probeDistances/blob/main/probeDist.m
2. Change the address field to the folder where the particle files are saved.
3. Modify the underlined section of the first line of code to load the particle file you wish to analyze:
  particles = readtable('____.csv')
4. Change “mkdir” to the worm and synapse you are analyzing, e.g., mkdir 3_1. Then, cd to that new directory.
5. Lines 4–9 set the x and y coordinates of each imaging channel. “particles.vis_probe == 0” is channel 1 in SRX, “==1” is channel 2, and “== 2” is channel 3. Keep this in mind when considering which channels to analyze. The script default is to compare channel 3 to channel 1.
6. Run each section of the code to measure the distances between the localizations of the first channel to the center of the second channel, and the localizations of the second channel to the center of the second channel.
7. Each section will output two variables called “dist”; these are your measurements.
*Note: After each section of code, save the “dist” distance outputs before analyzing a new set of channels. We recommend you plot the data in MATLAB. Other graphing software has a maximum number of points that can be displayed on a plot.*

**Counting calcium channels**

Foreword: Presented here are two methods to quantify the number of calcium channels at synaptic boutons. Both methods rely on clustering localizations into possible single protein domains. A caveat for both of these methodologies is that tight clusters of proteins can skew the estimate for blinks or for photon yield for a single protein. These methods of counting are more accurate for dispersed proteins than densely localized proteins but likely produce an undercount of the total number of proteins.

The methods differ in the quantification of signal within those protein clusters. The first calculates the number of localizations per protein. The second calculates the number of photons per protein. The advantages of one over the other are unclear; we recommend users attempt both methods and compare results. For a full discussion of counting methods refer to the methods section titled “Counting calcium channels” in Mueller et al. [14].

1. Counting proteins by position and particle count (Figure 5B and 5C):
  - Open the cluster file in Excel from section G in Microsoft Excel.
  - The ID column refers to cluster ID. Each row is a different cluster of localizations. Cluster 0 are single blinks. “particleCount” contains the number of localizations from channel 1 in each cluster. Using this information, we can calculate how many blinks each protein emitted.
  - Create a new Excel file called spreadsheet2. Name columns as Number of blinks, Count, Particles in Clusters, Total particles, and Single particles.
  - Create rows numbered 1 through the max number of “particleCount” in a cluster ID.
  - Manually count how many clusters contain each *number* of particles and input the total count in the “count” column. Do this for each synapse and replicate.
  - Combine these data across synapses or replicates and fit a Poisson distribution to the cumulative data. Lambda equals the mean number of blinks emitted from a protein in the dataset. Record this value on spreadsheet2.
  - Take the sum of the total number of localizations at each synapse and then divide the total number of blinks by the lambda value to yield how many proteins are present at each synapse. Plot the mean and SEM of these data. We recommend analyzing at least six synapses from three different animals.
2. Counting proteins by photon flux (Figure 5D and 5E):
  - Open the particle file from section G in Microsoft Excel.
  - Sort particles by clusterID. Note that clusterID = 0 are non-clustered localizations.
  - Sum the photons (“photon-count”) for each non-clustered localization and each clusterID.
  - Sum the total photons for all ClusterIDs.
  - Repeat 2a–2d for each synapse.
  - Take the average “photon-count” per cluster across the dataset.
  - Divide the “photon-count” from each synapse by the average “photon-count” per cluster (total photons divided by the average number of photons per channel). The result is the number of channels present at that synapse by photon flux. Plot the mean and SEM of these data. We recommend analyzing at least six synapses from three different animals.
*Note: We found that counting by photon flux had higher variability than counting by particle count; however, there was agreement between the results. An example of blink number and photon yield for clusters is present in Figure 5F.*
    **Figure 5.**
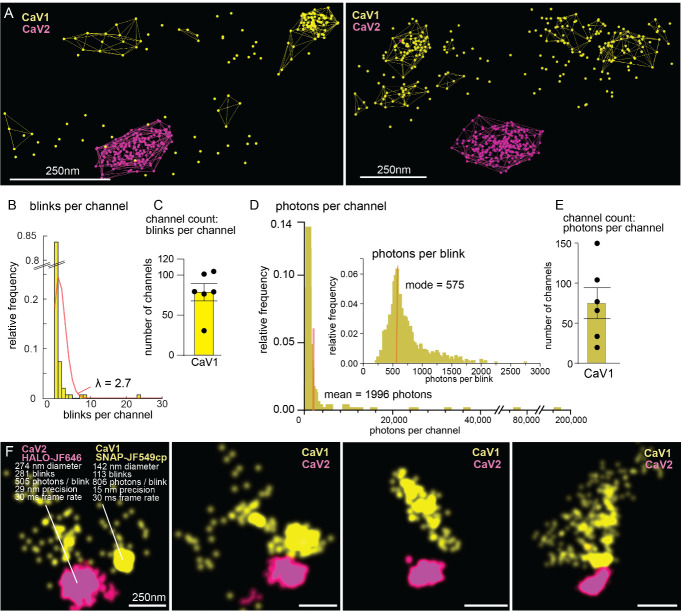
Counting calcium channels. (A) CaV2 (magenta) and CaV1 (yellow) localizations with cluster shells. (B–C) Quantification output from counting by particle count. Poisson curve fitting revealed that each protein blinked 2.7 times. (D–E) Quantification output from counting by photon flux. Each protein emitted on average 1996 photons. (F) Example statistics from voltage-gated calcium channel clusters at *C. elegans* synapses. Scale bar = 250 nm. Figure adapted from Mueller et al. [14].

**Data availability and example data**

All data mentioned in this study is available as source data files with the original publication. For ease of access, we have included particle files, cluster files, and channel count files as supplementary files with this protocol.

## Validation of protocol

This protocol or parts of it has been used and validated in the following research article(s):

- Mueller et al. [14]. CaV1 and CaV2 calcium channels mediate the release of distinct pools of synaptic vesicles. eLife (Figure 4, panels B, C; Figure 5, panels A–C; Figure 5 supplement 1, panels A–E; Figure 6, panels A–D; Figure 7, panels A–D; Figure 8, panels A–D; Figure 10, panels A–G).

## Supplementary information

The following supporting information can be downloaded here:

1. Cluster files
2. Particle files
3. Counting channels by photon flux.xlsx
4. Counting channels by position and particle count.xlsx
